# The impact of theory of mind, stress and professional experience on empathy in Romanian community nurses—a cross-sectional study

**DOI:** 10.1186/s12912-023-01569-2

**Published:** 2023-10-25

**Authors:** Lidia Onofrei, Costela Lacrimioara Serban, Adela Chirita-Emandi, Roxana Maria Jeleriu, Maria Puiu

**Affiliations:** 1 Department of Microscopic Morphology Genetics Discipline, Center of Genomic Medicine, Regional Center of Medical Genetics Timis, “Victor Babes” University of Medicine and Pharmacy Timisoara, 2 Eftimie Murgu Sqr, 300041 Timisoara, Romania; 2 Regional Center of Medical Genetics Timis, Clinical Emergency Hospital for Children “Louis Turcanu” Timisoara, Timis, Romania; 3 Department of Functional Sciences, Discipline of Public Health, “Victor Babeș” University of Medicine and Pharmacy Timisoara, 2 Eftimie Murgu Sqr, 300041 Timișoara, Romania

**Keywords:** Community nurses, Empathy, Theory of mind, Stress

## Abstract

**Background:**

High empathy levels in health professionals represent an important factor in patient satisfaction and compliance, reducing patient anxiety and pain, enhancing diagnostic and clinical results and strengthening patient empowerment. Our purpose was to determine empathy level and to identify which of the socioeconomic status (SES) and psychological factors were able to predict highest empathy levels in a Romanian sample of community nurses.

**Methods:**

Community nurses were invited in January-February 2023 to provide an answer to an online survey, using an advertisement in a professional network. 1580 participants voluntarily agreed to take part in this study, with a response rate of 85.8%. The survey included the Toronto Empathy Questionnaire, the Reading the Mind in the Eyes Test and socio-economic status items. A multivariate model for the prediction of belonging to the highest quartile of empathy as opposed to lowest quartile was constructed using SES and psychological variables as factors.

**Results:**

The mean (SD) empathy level was 49.1 (6.7), with 74.7% of participants over the threshold of high empathy level. In the multivariate analysis, predictors of belonging to the highest quartile of TEQ, as opposed to the lowest quartile were: low self-perceived stress level (OR = 2.098, 95%CI 1.362–3.231), higher experience as a community nurse (OR = 1.561, 95%CI 1.120–2.175) and higher levels of the theory of mind (OR = 1.158, 95%CI 1.118–1.199), when controlling for gender, age, relationship status, presence of children in families, education, and income.

**Conclusions:**

Training programs targeting to increase emotional competences, reduce levels of stress and encourage personnel retention have the potential to increase the quality of community nursing in Romania.

## Background

Individual emotional characteristics are inborn, however emotional competences can be taught and trained in familial environments and in schools [1]. While practiced in social and cultural contexts, emotional skills have a positive impact on workplace performance, interpersonal relations, stress management and promotion of healthy lifestyles [2].

Empathy is an important part of our human interaction, allowing the connection with others, with positive effects both in professional and personal life. Empathy is a critical skill in the medical profession, especially in nurses, because it is the base of good communication with patients. In a systematic review [3], empathy of health professionals was found to be an important factor in patient satisfaction and compliance, reducing patient anxiety and pain, enhancing diagnostic and clinical results and strengthening patient empowerment.

It has been reported that health professionals can be psychologically affected by dehumanization. Dehumanization was associated with several factors such emotional exhaustion and stress, as well as to work related factors such as staff ratios and patient care automation. The effect of dehumanization on health professionals is determining them to practice medicine mechanically, with moral disengagement and without empathy [2, 4, 5] .

It is considered that adequate empathic social behavior is due to the perfect balance between mentalizing, involving cognitive and affective theory of mind and simulation processing, represented by emotional empathy. Since these emotional experiences are mediated by different neural networks: amygdala and insula for emotional experiences and ventromedial prefrontal cortex for the theory of mind, it has been proposed that empathy and the theory of mind are multidimensional [6].

Since aspects of empathy have not been measured in a Romanian national representative sample of community nurses so far, our research hypothesis was to determine empathy level and to identify which of the socioeconomic status (SES) and psychological factors were able to predict highest levels of empathy in this cohort.

## Methods

### Participants

Community nurses provide medical and nursing care to patients outside of traditional hospital settings. In Romania, their aim is to increase the access of the population and, in particular, of vulnerable groups to quality medico-social services. Their job includes preventive, curative, and recovery medical services, including case management in complex cases of chronic diseases and rare diseases. Of 1840 community nurses employed in Romania in January—February 2023, 1580 participants agreed to take part in this study, with a response rate of 85.8%.

### Procedure

The sample was recruited by an advertisement in a professional network in January—February 2023. The project was shortly advertised on a national work platform for community nurses from Romania to participate in a research project. The questionnaire was set online on the Google Forms platform and the link to the questionnaire was provided in the advertisement. The first page of the questionnaire included the purpose of the study, the estimated completion time, and some information on the instrument used and the result of the questionnaire. The agreement to participate was included in this introductory page and only those who gave their consent were allowed to access the questionnaire. All questions were required and participants who provided answers to all questions were allowed to submit the form. As retribution for their involvement, at the moment of questionnaire completion, the participants received a report describing their perception of emotions ability. The Research Ethics Committee of Victor Babes University of Medicine and Pharmacy approved the study protocol (no Nr. 30/31.03.2022). Participants voluntarily agreed to participate and gave informed consent. The study was conducted in accordance with the Helsinki declaration.

### Materials

#### Toronto Empathy Questionnaire (TEQ)

TEQ was developed by Spreng et al. [7] and is a self-report questionnaire measuring a person’s emotional ability to understand and respond to others. This test includes 16 questions, half of which are positively worded (items 1, 3, 5, 6, 8, 9, 13, 16) and the other half are negatively worded (items 2, 4, 7, 10, 11, 12, 14, 15). For each item, answers must be chosen from the following options: Never; Rarely; Sometimes; Often; Always. Positive items are scored Never = 0; Rarely = 1; Sometimes = 2; Often = 3; Always = 4 and negative items are scored Never = 4; Rarely = 3; Sometimes = 2; Often = 1; Always = 0. Total TEQ score was obtained by adding the individual item scores. For this study, the validated Romanian version [8] of TEQ was used. The score ranges from 0 to 64. Individuals scoring 45 or higher are considered with high levels of empathy.

#### The ‘reading the mind in the eyes’ test (RMET)

This test includes 36 black and white photographs of actors, taken from movies, of male and female eyes depicting different emotional states. Participants are asked to choose the best description of four possible emotions that fits the eyes’ emotions. For this test, the performance of the participants was calculated as the total points of correct answers of 36 images. We used the Romanian version of the Reading the Mind in the Eyes Test [9].

#### SES related questions

Besides the questionnaires described above, the survey also included questions about gender, age, relationship status, presence of children in family, school (3 levels, equivalent to ISCED3 or 4, ISCED 5 or 6, ISCED 7 or 8), income (5 levels), years of experience as a community nurse and self-perceived stress levels. Stress levels were quantified on a 10 points scale, with higher levels indicating higher levels of stress.

### Data management and statistics

The questionnaire was set online using the Google Forms platform and the invitees were provided a link to the questionnaire. The first section of the questionnaire included a presentation of the survey and a consent form. IBM SPSS version 21 was used to perform data transformations and statistics. Kolmogorov-Smirnoff test was used to test normal distribution, and the main outcome variables (scores of TEQ and RMET) were all normally distributed. Continuous variables are presented as mean and standard deviation (SD). The categorical variables are presented as absolute and relative frequencies. A *p*-value < 0.05 was considered statistically significant. To compare means, the t-test and ANOVA were used. SES and psychological characteristics of community nurses were used to predict belonging to the highest quartile, as compared to the lowest quartile of the TEQ score, in a regression analysis model.

## Results

The demographic characteristics of the study population are presented in Table 1. Women represented 93.9% (1483) and the mean (SD) age of the group was 42.8 (8.2) years with a range from 22 to 65. Most of the responders, 85.3% (1347) are in a relationship and 86.3% (1364) have at least one child, while 21.7% (343) of them reported having at least a university degree—The International Standard Classification of Education (ISCED 5). On the experience as a community nurse, 46.1% (511) have experience of 5 years or less and more than half of all 58.2% (920) declare income below 3000 lei (equivalent to 611 euro, the minimum income level in January 2023 in Romania). 13.4% (211) have quantified their stress level as 7 points or more out of a scale of 10, qualifying as high levels of stress. For RMET, the mean score was 22.1 (4.7).

**Table 1:** SES characteristics of the sample (N = 1580)

Factors		Count (%)
Gender	Male	97 (6.1%)
	Female	1483 (93.9%)
Age	Under 40 years	566 (35.8%)
	40–50 years	744 (47.1%)
	Over 50 years	270 (17.1%)
	Mean (SD)	42.8 (8.2)
In a relationship	No	233 (14.7%)
	Yes	1347 (85.3%)
How many children they have	None	216 (13.7%)
	1 child	563 (35.6%)
	2 children	675 (42.7%)
	3 or more children	126 (8.0%)
Education	High School or post-high school diploma (ISCED 3 or 4)	1237 (78.3%)
	College or university diploma (ISCED 5 or 6)	282 (17.8%)
	Master’s degree or more (ISCED 7 or 8)	61 (3.9%)
Employment as a community nurse	5 years or less	683 (43.2%)
	6 or more years	897 (56.8%)
	Mean (SD)	9.1 (6.2)
Income	Under 3000 RON (611 Euro)	920 (58.2%)
	3000 RON (611 Euro) or above	660 (41.8%)
High self-perceived stress level	Yes	211 (13.4%)
	No	1369 (86.6%)
RMET	mean (SD)	22.1 (4.7)

ISCED—International Standard Classification of Education, RON—Romanian Leu, RMET—Reading the Mind in the Eyes’ test, SES—socioeconomic status, SD—standard deviation

The mean scores for TEQ by SES factors are presented in Table 2. For TEQ, the mean score was 49.1 (6.7). The score was significantly higher 49.5 (6.6) for community nurses with more than 6 years of experience, as compared to those with 6 years or less 48.6 (6.8). No significant difference was found for the other SES factors tested: gender, relationship status, presence of children, education, income and age categories. Also, TEQ levels were significantly higher in participants reporting low levels of stress 49.5 (6.4), as compared with those reporting high levels of stress 46.1 (7.7). Per tertiles of RMET score, a significant positive trend is observed, and correlation between RMET score and TEQ score r = 0.282, p < 0.001, sharing 8% of variance.

**Table 2:** Mean (SD) values of TEQ for SES factors

SES factors		TEQ	p-value*
		Mean Standard Deviation	
Gender*	Male	48.7 (5.9)	0.677
	Female	49.1 (6.8)	
Age categories*	Under 40 years	48.7 (7.1)	0.105
	40–50 years	49.1 (6.6)	
	Over 50 years	49.7 (6.3)	
In a relationship*	No	48.8 (7.3)	0.599
	Yes	49.1 (6.6)	
Children*	no	48.6 (6.8)	0.362
	yes	49.1 (6.7)	
Education*	ISCED 4 or less	48.9 (6.7)	0.181
	ISCED 5 or more	49.5 (6.7)	
Experience as community nurse**	First tertile	48.5 (6.6)	0.045
	Second tertile	48.9 (6.6)	
	Third tertile	49.6 (6.5)	
Income*	Under 3000 RON (611 Euro)	49.0 (6.8)	0.629
	3000 RON (611 Euro) or above	49.2 (6.7)	
High self-perceived stress level*	Yes	49.5 (6.4)	< 0.001
	No	46.1 (7.7)	
RMET **	First tertile	46.5 (6.9)	< 0.001
	Second tertile	50.0 (6.1)	
	Third tertile	50.9 (6.1)	

ISCED—International Standard Classification of Education, RON—Romanian Leu, RMET—Reading the Mind in the Eyes’ test, SES—socioeconomic status, SD—standard deviation, TEQ—Toronto Empathy Questionnaire, *T-test **ANOVA p-values < 0.05 are considered significant

Using only participants from the lowest and highest quartile of TEQ scores, we created a prediction model using SES and psychological variables. The OR and the 95% confidence intervals of the OR of each predictor fitted into the model are presented in Table 3. Highest OR (2.098, 95%CI 1.362–3.231) to pertain to the top quartile of TEQ was achieved by the low levels of stress. Higher experience as a community nurse had the OR = 1.561, 95%CI 1.120–2.175. RMET score contributed to the model with an OR = 1.158, 95%CI 1.118–1.199. The other social demographic factors did not significantly contribute to the model, yet are accounted for in the model.

**Table 3:** SES predictors of belonging to highest quartile of TEQ as compared to lowest quartile

SES factors	OR	95% confidence interval for OR	
Male gender	0.761	0.376	1.541
Age (years)	0.990	0.969	1.011
In a relationship	0.779	0.502	1.208
At least one child	1.092	0.677	1.760
ISCED 4 or less	0.930	0.636	1.358
6 or more years of experience as a community nurse	1.561	1.120	2.175
3000 RON (611 Euro) or above	0.939	0.679	1.299
Low levels of stress (6 points or less out of 10 points)	2.098	1.362	3.231
RMET score	1.158	1.118	1.199

ISCED—International Standard Classification of Education, RON—Romanian Leu, RMET—Reading the Mind in the Eyes’ test, SES—socioeconomic status, SD—standard deviation TEQ—Toronto Empathy Questionnaire, 611 euro—the minimum wage in January 2023

Dependent variable: highest vs. lowest quartile of TEQ; Independent variables: gender (2 categories), Age (years), Relationship status (2 categories), Children (2 categories), education (2 categories), experience (2 categories), income (2 categories), Self-perceived stress levels (2 categories), RMET score (discrete units of score).

## Discussion

The nurse-patient relationship is modulated by emotional competences. To be empathetic to patients and to have the capacity to read emotions is a fundamental aspect of care. These capacities can be regulated by other emotional abilities as well as technical skills in order to acknowledge the patient’s needs and offer therapeutically appropriate response [10]. To the best of our knowledge, empathy was not studied in a Romania national sample of community nurses. Our study improves the understanding of the emotional competences of this professional group.

As required by roles played by these professionals, the mean levels of empathy are increased, with 74.7% of the sample being over the threshold of high empathy level (scoring > = 45), while the mean (SD) reported score was 49.1 (6.7). A recent study investigating the empathy among senior medical students from Romania has found a similar mean (SD) level of empathy of 48.76 (5.65) [8]. Using the same instrument, when studying nurses that work in psychiatric wards, Alhadidi et al. [11] have identified lower levels of empathy, reporting a mean (SD) of 46.07 (6.7). Conversely, Cosper [12] found similar levels of empathy 49.8 (4.3) in nurses working in the Intensive Care Unit (ICU).

Our results indicate that as experience as a community nurse accumulates, the empathy level increases: nurses with more than 6 years of experience had 56% more chances to be in the higher quartile of empathy, suggesting that job or field retention is a key factor for increased empathy. Observing the tertiles of years of experience, the empathy level increases steadily. A publication on the 10 year trend of empathy in intensive care units from China [13] found a reduction in empathy levels. Cosper et al. [12] did not identify a significant association between empathy level and years of experience as a nurse, however found a negative correlation with the time spent as a ICU nurse. For nurses working in oncology, increasing experience was related to reduced capacity to verbally respond and listen to the patients, perhaps as their roles and the need to learn technical skills were expanding [14]. A longitudinal study [15] that included nursing students involved as part of their curricula in different aspects of training, found that nursing students that had more clinical encounters with patients had a decline in empathy levels after 1 year of follow-up, while students with less clinical encounters did not significantly modify the empathy during the evaluation. A cross-sectional study [16] has similar results, reporting a drop in empathy in 4th year students, as compared to students from the first year. This erosion of empathy in the clinical setting was attributed to patients’ negativity and time restricted interaction between nurses and patients [17, 18]. Others [19] have found that among emergency department nurses, low level of manager support was a significant predictor of higher levels of burnout and compassion fatigue. Using a subgroup analysis among master nursing students, Hakansson Eklund et al. found different empathy levels, with public-health nursing, midwifery and psychiatry students scoring higher than anesthesia and intensive care master students [20].

Similarly, studies performed in medical doctors have shown that empathy levels decrease over time due to a coping mechanism for burnout, in association with depressive symptoms [21]. In medical students, several studies have discovered a decline in empathy at the beginning of the clinical phase of medical education [18, 22, 23]. Several studies looked into the reasons for this decline and found that its coping mechanism for distress [24] and was caused by school related factors such as lack of time, physical and psychological fatigue, competitiveness, performance demands, cramming, stress, high workload [25, 26], some of these factors belonging to the hidden curriculum to become a medical professional [27].

Possible explanations on the differences between our study and studies on nurses working in high demanding positions like in ICU, oncology or psychiatry or while in training, lie in the job description of community nurses, which are engaged in a diverse and challenging health landscape, providing care is different settings from patient’s own houses, nursing homes, family doctor practices, or other types of clinics.

High levels of self-perceived stress are a good predictor of low levels of empathy, both in univariate and multivariate models. Others have also reported negative associations between stress/anxiety and empathy, with stress/anxiety management having a positive effect on empathy levels [2, 28, 29]. Extensive prior research [30] has shown how anxiety reduces empathy, suggesting that experiencing emotions associated with uncertainty, increases egocentric perspectives when perceiving the mental states of others.

Cognitive empathy—the capacity to understand other people by ascertaining their mental state, also known as the theory of mind, quantified in our study by the RMET, shows a positive association with the TEQ score, sharing 8% of variance. In the multivariate model, by each one point increase in the RMET score, the chances to be in the high quartile of TEQ as opposed to the low quartile grow by 16.5%. In a recent study [2] on nurses, cognitive empathy was a mediator between emotional intelligence and humanization of care, with 18% shared variance between cognitive empathy and humanization of care. In another study [31], performed in general population, using a prediction model for the RMET score by components of the emotional intelligence, have found that the highest contributor is the understanding subscale (β = 0.28, p < 0.001), followed by perceiving (β = 0.09, p < 0.01), and managing subscales (*β* = 0.08, *p* < 0.05). The variance shared between emotional and cognitive intelligence was 8.4%, similar to our results. Using subgroup analysis from the general population, Baron-Cohen et al. [32] have found lower shared variance, at about 2%, between cognitive empathy and empathy quotient.

Our study did not find significant differences in empathy levels between genders in this population, similar to several studies on nursing students [13] or psychiatric ward nurses [11] or ICU nurses [12]. Also, education level, familial background and income levels did not influence the empathy level in our sample. Others have found that empathy was higher in nurses with higher education, albeit those nurses were working in ICU [33] or were students with a previous degree before enrolling in studying nursing [34]. Studies performed to capture empathy in family dynamics have shown that positive family environments are positively related to empathy levels in adolescents [35]. In a longitudinal study [36], high compassion for others in adulthood predicted, after a 10-year follow-up, higher adulthood social-economic status, although not vice versa.

Considering that high levels of empathy are critically needed for nursing, it is important to acknowledge that empathy can be learned and taught. Making a review of European training programs for emotional competencies in vocational training, Sauli et al. [1] have found four types of training available in Europe, however they lacked a rigorous scientific approach, proposing a set of guidelines for program development. Using an educational program designed by patient and family advisers, Cosper [12] demonstrated an increase in empathy levels of nurses, higher in those younger than 30 years, as compared to their counterparts. Thus recognizing the benefit of early career educational programs.

Two of the strengths of this study are the large number of participants and the high response rate of 85.8%, leading to the assessment of a representative national sample of community nurses from Romania. Looking into the response rate of different organizational research based on questionnaires, Baruch et al. [37] found that the response rate in public/state sector varied from a minimum of 27.0% to a maximum of 82.8% with a mean (SD) of 54.5 (16.7) and in Health care from a minimum of 17.4% to a maximum of 94.0% with a mean (SD) of 53.8 (20.0). By comparison, our results sit in the highest quartile. If this high response rate, which was obtained without reminders is used as a proxy for empathy, the result is concordant to high scores in TEQ, where 74.7% of responders had a high empathy score. If we would use this proxy, it might be that the non-responders would have lower empathy levels, inducing a selection bias, because people interested in participation usually have a genuine interest in the subject of the research.

## Conclusion

The analysis of empathy in a large sample of Romanian community nurses showed good levels of empathy, since almost three quarters of them scored high empathy levels. The capacity of reading emotions (theory of mind abilities) and higher experience, along with low levels of stress, led to higher levels of empathy. Future early-career training programs targeting to increase emotional competences, reduce levels of stress and encourage personnel retention could promote increased quality of community nursing in Romania.

## Data Availability

The datasets used and/or analyzed during the current study are available from the corresponding author on reasonable request.
